# Evaluation of Perennial *Glycine* Species for Response to *Meloidogyne Incognita*, *Rotylenchulus Reniformis*, and *Pratylenchus Penetrans*

**DOI:** 10.21307/jofnem-2022-001

**Published:** 2022-02-18

**Authors:** Jaeyeong Han, Steven P. Locke, Theresa K. Herman, Nathan E. Schroeder, Glen L. Hartman

**Affiliations:** 1 Department of Crop Sciences, University of Illinois, Urbana, IL 61801.; 2 USDA-Agricultural Research Service, Urbana, IL, 61801.

**Keywords:** *Meloidogyne incognita*, perennial Glycine, *Pratylenchus penetrans*, resistance, *Rotylenchulus reniformis*

## Abstract

Root-knot (*Meloidogyne incognita* (Kofoid & White) Chitwood), reniform (*Rotylenchulus reniformis* Lindford & Oliveira), and lesion nematodes (*Pratylenchus penetrans* (Cobb) Filipjev & Schuurmans Stekhoven) are plant-parasitic nematodes that feed on soybean (*Glycine max* (L.) Merr.) roots, limiting seed production. The availability of resistance in soybeans to these nematodes is limited. However, new sources of resistance can be discovered in wild relatives of agronomic crops. Perennial *Glycine* species, wild relatives to soybean, are a source of valuable genetic resources with the potential to improve disease resistance in soybean. To determine if these perennials have resistance against nematodes, 18 accessions of 10 perennial *Glycine* species were evaluated for their response to *M. incognita* and *R. reniformis*, and eight accessions of six perennial *Glycine* species were evaluated for their response to *P. penetrans*. Pot experiments were conducted for *M. incognita* and *R. reniformis* in a growth chamber and in vitro experiments were conducted for *P. penetrans*. We found both shared and distinct interactions along the resistance-susceptible continuum in response to the three plant-parasitic nematode species. Ten and 15 accessions were classified as resistant to *M. incognita* based on eggs per gram of root and gall index, respectively. Among them, *G. tomentella* plant introductions (PIs) 446983 and 339655 had a significantly lower gall index than the resistant soybean check cv. Forrest. Of three *R. reniformis* resistant accessions identified in this study, *G. tomentella* PI 441001 showed significantly greater resistance to *R. reniformis* than the resistant check cv. Forrest based on nematodes per gram of root. In contrast, no resistance to *P. penetrans* was recorded in any perennial *Glycine* species.

Southern root-knot nematode (*Meloidogyne incognita* (Kofoid & White) Chitwood), reniform nematode (*Rotylenchulus reniformis* Linford & Oliveira), and lesion nematode (*Pratylenchus penetrans* (Cobb) Filipjev & Shuurmans Stekhoven) are common plant-parasitic nematodes that infect soybean (*Glycine max* (L.) Merr.) and other crops, causing yield losses (Noel et al., 2015; Bradley et al., 2021). These nematodes occur in diverse soybean growing regions (Karssen et al., 2013; Noel et al., 2015). Yield loss caused by plant-parasitic nematodes including *Meloidogyne* spp., *R. reniformis,* and *Pratylenchus* spp. in the United States and Ontario, Canada in 2019 was estimated at 366,647 metric tons (Bradley et al., 2021). *M. incognita* was the second most damaging pathogen in the southern United States in 2019 (Bradley et al., 2021) and can cause up to 90% yield reduction on susceptible soybean cultivars (Kinloch, 1974). *R. reniformis* can cause 30–60% yield loss depending on the soybean cultivar (Noel et al., 2015). *Pratylenchus* spp. cause dark lesions on soybean roots reducing root mass by 25% (Ferris and Bernard, 1962).

Host resistance is an important management strategy for controlling plant-parasitic nematodes on soybean. For example, plant introduction (PI) 88788 is the most commonly used source of resistance in soybean against the soybean cyst nematode (SCN, *Heterodera glycines* Ichinohe) (Faghihi et al., 2010; McCarville et al., 2017). However, soybean fields planted with the SCN-resistant soybean cultivars may be vulnerable to attack by other nematodes including *M. incognita* and *R. reniformis* (Robbins and Rakes, 1996; Klepadlo et al., 2018). Among 76 soybean accessions with PI 88788-derived SCN resistance, 72% and 50% were susceptible to *M. incognita* and to *R. reniformis*, respectively (Klepadlo et al., 2018).

Numerous soybean germplasm accessions and cultivars were screened for resistance to *Meloidogyne* spp. and *R. reniformis* and resistant soybean lines have been identified (Rebois et al., 1968; Birchfield and Brister, 1969; Luzzi et al., 1987; Hussey et al., 1991; Robbins and Rakes, 1996; Robbins et al., 1999; Harris et al., 2003; Stetina et al., 2014; Klepadlo et al., 2018). Biparental linkage mapping and genome-wide association studies showed that resistance to *M. incognita* and *R. reniformis* in soybean is a quantitative trait (Williams et al., 1981; Tamulonis et al., 1997; Li et al., 2001; Ha et al., 2007; Pham et al., 2013; Xu et al., 2013; Jiao et al., 2015; Passianotto et al., 2017; Li et al., 2018; Wilkes et al., 2020). However, resistance mechanisms and associated resistance genes are poorly understood. For *P. penetrans*, no resistance has been identified in soybean, despite several efforts (Schmitt and Barker, 1981; Melakeberhan, 1998).

Wild relatives of domesticated crops may have unique disease resistance traits absent from modern-day crop varieties. For example, *M. incognita* resistance genes (*Mi* genes) originated from wild tomato relative *Solanum peruvianum* (Smith, 1944) and have been introgressed into many modern tomato varieties (*S. lycopersicum* L.) (Williamson, 1998). Wild perennial *Glycine* species are taxonomically and genetically related to soybean. Primarily originating from Australia, there are currently 27 described species of perennial *Glycine* (Barrett and Barrett, 2015; Singh, 2019). Perennial *Glycine* accessions have resistance to SCN (Riggs et al., 1998; Bauer et al., 2007; Wen et al., 2017; Herman et al., 2020). We hypothesize that resistance to other nematodes also exists in perennial *Glycine*. The objective of this study was to evaluate 18 accessions of 10 perennial *Glycine* species against *M. incognita* and *R. reniformis*, and eight accessions of six species against *P. penetrans*. To ensure diverse genetic representation, we selected SCN-susceptible and resistant accessions identified previously (Wen et al., 2017); *G. latifolia* (PI 559298 and PI 559300) and *G. tomentella* PI 505214 were chosen because of the availability of sequence information (Liu et al., 2018).

## Materials and methods

### Plant preparation

Eighteen perennial *Glycine* species and five soybean cultivars were obtained from the USDA-ARS Soybean Germplasm Collection (https://www.ars-grin.gov/) (Table 1). All accessions were originally collected from Australia except *G. tabacina* PI 446974 (Okinawa, Japan) and two *G. tomentella* accessions, PIs 446983 (Papua New Guinea) and 339655 (Taichung, Taiwan). Chromosome numbers varied from 40 to 80 (USDA-ARS Germplasm Resources Information Network, https://www.ars-grin.gov/).

**Table 1:** Accessions of perennial *Glycine* species and soybean checks inoculated with *Meloidogyne incognita*, *Rotylenchulus reniformis*, and *Pratylenchus penetrans.*

***Glycine* species**	**Accession**	**Origin**	**Chromosome number**
*G. argyrea*	PI 509451	Queensland, Australia	40
*G. canescens*	PI 573045	Western Australia, Australia	40
*G. canescens*	PI 440932	South Australia, Australia	40
*G. clandestina*	PI 440960	New South Wales, Australia	40
*G. cyrtoloba*	PI 509472	Queensland, Australia	40
*G. curvata*	PI 505167	Queensland, Australia	unknown
*G. latifolia*	PI 559298	Queensland, Australia	40
*G. latifolia*	PI 559300	Queensland, Australia	40
*G. microphylla*	PI 509487	New South Wales, Australia	40
*G. microphylla*	PI 505188	Queensland, Australia	40
*G. pescadrensis*	PI 505197	Queensland, Australia	80
*G. tabacina*	PI 446974	Okinawa, Japan	80
*G. tabacina*	PI 373990	New South Wales, Australia	40
*G. tomentella*	PI 446983	Papua New Guinea	40
*G. tomentella*	PI 505214	Queensland, Australia	80
*G. tomentella*	PI 339655	Taichung, Taiwan	80
*G. tomentella*	PI 441001	Queensland, Australia	78
*G. tomentella*	PI 505238	Queensland, Australia	80
*G. max* cv. Pickett71^a^	PI 548982	USA	40
*G. max* cv. Forrest^b^	PI 548655	USA	40
*G. max* ^c,d^	PI 88788	Liaoning Sheng, China	40
*G. max* cv. Lee 68^e^	PI 559369	USA	40
*G. max* cv. Williams 82^d^	PI 518671	USA	40

Notes:

a Susceptible check for *M. incognita* (Vanderspool et al., 1994).

b Resistant check for *M. incognita* and *R. reniformis* (Hussey et al., 1991; Robbins et al., 1994).

c Susceptible check for *R. reniformis* (Robbins and Rakes, 1996).

d Included in *P. penetrans* tests.

e Susceptible check for *P. penetrans* (Schmitt and Barker, 1981).

Perennial *Glycine* seeds were scarified with a razor blade by slightly cutting the seed coat on the opposite side of the hilum. Seeds were germinated for 5 to 7 days on wet tissue paper in a plastic box for *M. incognita* tests and on Sun Gro^®^ Sunshine^®^ LC1 Grower Mix (BFG Supply, Burton, OH) in petri dishes for *R. reniformis* tests. Seedlings were planted in steam pasteurized torpedo sand, for *M. incognita,* or sandy loam (77% sand, 11% silt, and 12% clay), for *R. reniformis,* in SC10 Conetainers (Stuewe and Sons, Tangent, OR). Three weeks after planting, the seedlings were inoculated with nematodes. Seeds of susceptible (cv. Pickett 71 for *M. incognita*; PI 88788 for *R. reniformis*) and resistant soybean checks (cv. Forrest) were germinated following the same methods above (but not scarified) and planted in torpedo sand or sandy loam 1 week prior to inoculation.

For *P. penetrans*, root explants were prepared on agar media. Scarified perennial *Glycine* seeds were surface disinfected in 0.5% sodium hypochlorite (NaOCl) for 5 min and rinsed three times with sterilized distilled water. Susceptible check cv. Lee 68 and other soybeans, PI 88788 and cv. Williams 82 were surface disinfected in 0.5% NaOCl for 20 min and rinsed three times with sterilized distilled water. Five seeds of each accession were transferred onto a Murashige and Skoog (MS) (Murashige and Skoog, 1962) solid medium supplemented with 2% sucrose for germination. Seeds were incubated in a growth chamber at 25°C with 16 hr of fluorescent light per day for nine days. Germinated seedlings were transferred to new MS medium supplemented with 2% sucrose (one plant per plate) for inoculation.

### Nematode source and plant inoculations

*M. incognita*, originally isolated from soybean in southern Illinois (generous gift from Jason Bond) and identified using polymerase chain reaction (PCR) with species-specific primers (Adam et al., 2007), was maintained on tomato (*S. lycopersicum*) cv. Tiny Tim in the greenhouse. Tomato roots with root galls were cut into small pieces and mixed with 200 ml of 0.5% NaOCl and vigorously shaken manually for 4 min to release eggs from the gelatinous matrix (Hussey and Barker, 1973). The mixture was filtered through 74- and 25-μm sieves and thoroughly rinsed with tap water. Eggs were centrifuged in 45.4% sucrose solution to remove plant and soil debris (Jenkins, 1964). Perennial *Glycine* and soybean seedlings were inoculated with 2,000? *M. incognita* eggs in 1 ml of water per plant into a 2.5-cm deep hole made 1.5-cm away from each stem.

*R. reniformis*, originally isolated from a cotton (*Gossypium hirsutum* L.) in College Station, Texas, was maintained on soybean cv. Macon or cv. Braxton in the greenhouse (generous gift from Martin Wubben). To extract vermiform *R. reniformis*, the roots were removed, soil was suspended in water, and poured through 841- and 38-μm sieves (Robbins et al., 1999). Extracted nematodes on the 38-μm sieve were placed on a Baermann funnel to collect live nematodes after 24 hr. Perennial *Glycine* and soybean seedlings were inoculated with 1,000 mixed stage nematodes in 1 ml of water per plant into a 2.5-cm deep hole made 1.5-cm away from each stem.

*P. penetrans*, originally isolated from potato in Rosholt, Wisconsin (*Solanum tuberosum* L.) and identified based on morphological characteristics and mitochondrial cytochrome c oxidase subunit 1 and 28S rDNA sequences (Saikai and MacGuidwin, 2020), was maintained on monoxenic corn root cultures (Rebois and Huettel, 1986). The root cultures were cut and immersed into sterilized distilled water in a beaker and shaken at 75 RPM for 24 hr at room temperature to suspend nematodes into water. The suspension was poured onto an autoclaved hatching chamber in a plastic box to collect live nematodes in a sterile condition (Thapa et al., 2017). Live nematodes were collected after 24 hr at room temperature. Perennial *Glycine* and soybean seedlings were inoculated with 150 mixed stages nematodes of *P. penetrans* in 50 μl of sterilized distilled water per plant.

### Experimental design

All tests were conducted in a completely randomized design (CRD) with five replications and each test was repeated once. Data were not collected from a few experimental units where seeds did not germinate or where seedlings were too small to inoculate. *M. incognita* and *R. reniformis* tests were conducted in a growth chamber at 28°C and 16 hr of fluorescent light per day for 8 weeks for the *M. incognita* test and 10 weeks for the *R. reniformis* test. Plants were fertilized with a 100-ppm solution general purpose fertilizer (Peter's Professional 20-20-20) weekly after transplanting. Two soybean genotypes for susceptible and resistant checks were included in each experiment (Table 1). The susceptible and resistant checks were selected based on previous research (Luzzi et al., 1987; Hussey et al., 1991; Robbins et al., 1994; Vanderspool et al., 1994; Robbins and Rakes, 1996; Allen et al., 2005). *R. reniformis* infested fallow soil was included in the test as a survival baseline control without host (Robbins and Rakes, 1996; Robbins et al., 1999).

*P. penetrans* tests were conducted in a growth chamber at 25°C and 16 hr of fluorescent light per day for six days. *G. max* cv. Lee 68 was included as a susceptible check (Schmitt and Barker, 1981). *G. max* cv. Williams 82 and PI 88788 were also included to examine their response to *P. penetrans*. No resistant soybean checks were included in this study because there are no resistant checks known for soybean.

### Nematode response evaluation

The response to *M. incognita* was recorded based on the number of eggs per gram of fresh roots and gall index (the extent of root galling) 8 weeks after inoculation (Taylor and Sasser, 1978; Bridge and Page, 1980). Plant roots were washed to remove sand and weighed. The gall index was assessed based on the root-knot rating chart (Bridge and Page, 1980). *M. incognita* eggs were extracted from whole root systems as described above and enumerated under a dissecting microscope at ×50 magnification.

The response to *R. reniformis* was evaluated using the final number of nematodes (eggs and vermiform) per gram of fresh root 10 weeks after inoculation. To extract *R. reniformis*, soil was washed from the plant root, suspended in water, and poured through 250- and 38-μm sieves. Nematodes collected on the 38-μm sieve were further processed by sucrose-centrifugation (Jenkins, 1964). *R. reniformis* eggs were extracted from the roots as described above after measuring the fresh weight of the washed plant roots.

The response to *P. penetrans* was based on nematode counts in the roots following acid fuchsin staining (Bybd et al., 1983). Seedlings were removed from media six days after inoculation and cut below cotyledons. Roots were weighed and stained with acid fuchsin. Nematodes in the stained roots were enumerated under a dissecting microscope and reported as nematodes per gram of root.

### Data analysis

To determine if trials within each test could be combined, homogeneity of variance was determined by the Bartlett test using JMP Pro 14.2.0 Fit X by Y platform (SAS Institute, Cary, NC). The analysis of variance (ANOVA) was done for each trial individually and for both tests pooled if homogeneity of variance was not significant between trials. *M. incognita* eggs per gram of root data and *P. penetrans* nematodes per gram of root were log (x + 1) transformed and *R. reniformis* nematodes per gram of root data were log (x) transformed to meet normality and homogeneity of variance assumptions. The ANOVA analyses were done with JMP Pro 14.2.0 Fit X by Y or Fit Model platforms. Mean separations were done using JMP Pro 14.2.0 Tukey-Kramer HSD test at *α* = 0.05 (Dunnett, 1980).

### Resistance rating

Nematode resistance levels in perennial *Glycine* species were categorized as susceptible (S), moderately resistant (MR), and resistant (R) based on the statistical comparison with susceptible and resistant soybean checks: S ≥ susceptible check; susceptible check > MR > resistant check; R ≤ resistant check. Ratings were determined by combining trial data for both *M. incognita* and *R. reniformis,* while *P. penetrans* trial data were kept separate.

## Results

### Evaluation of perennial *Glycine* species for resistance to *M. incognita*

**Figure 1:**
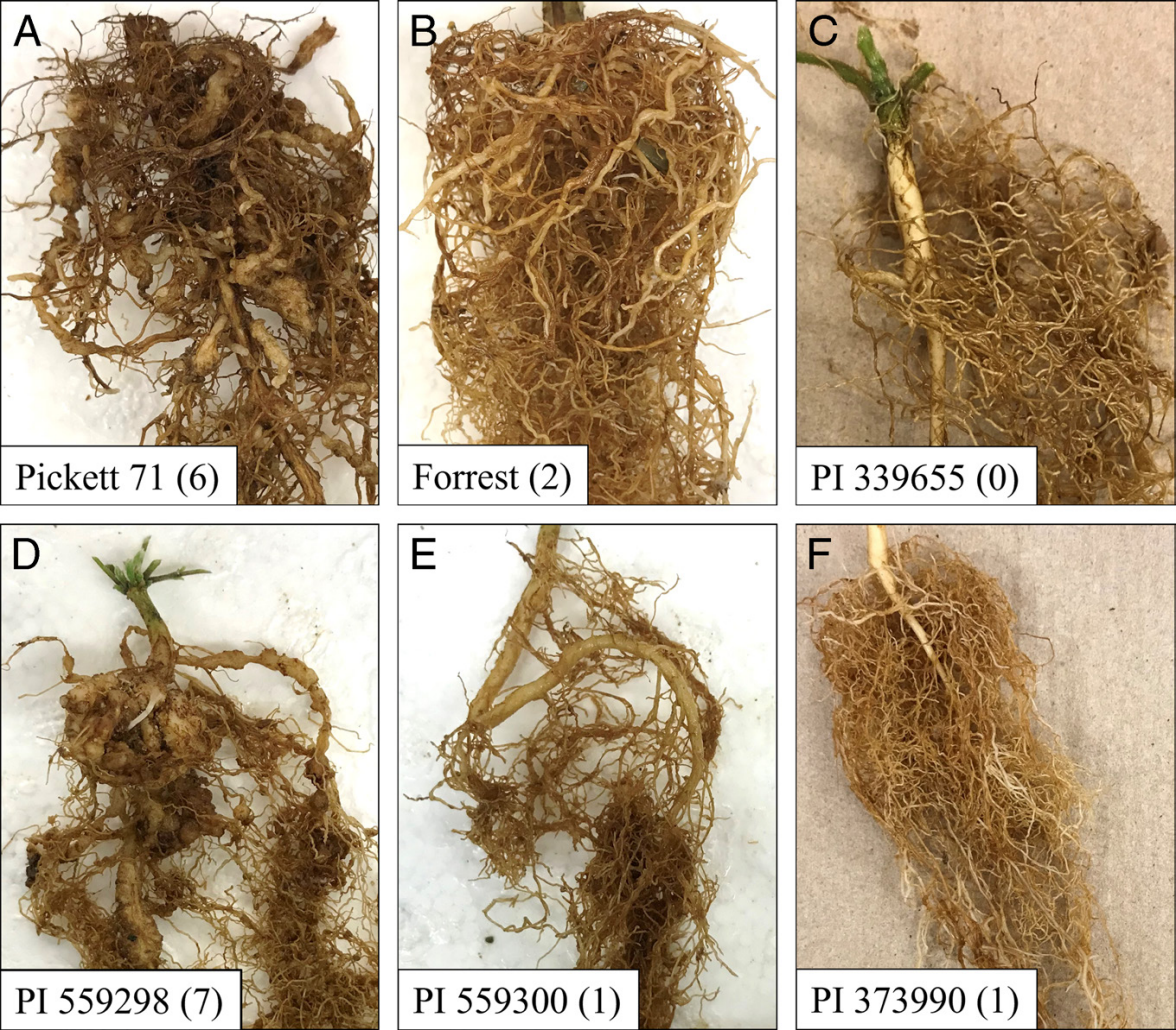
Representative images of soybean and perennial *Glycine* species roots at 8 weeks post-inoculation of *Meloidogyne incognita*. Gall index rating is given in parentheses A, susceptible check *G. max* cv. Pickett 71. B, resistant check *G. max* cv. Forrest. C, *G. tomentella* PI 339655. D, *G. latifolia* PI 559298. E, *G. latifolia* PI 559300. F, *G. tabacina* PI 373990.

The Bartlett tests for homogeneity of variance for eggs per gram of root and gall index were not significant (*P* > 0.05) between trials, so data were pooled for analysis (Table 2). Our results demonstrate substantial variation in nematode reproduction and gall index among perennial *Glycine* accessions (Table 2 and Fig. 1). The susceptible check soybean cv. Pickett 71 was not significantly different from the resistant check cv. Forrest in eggs per gram of root but had a significantly greater gall index (Table 2; Fig. 1A, B). Ten perennial *Glycine* accessions (PIs 373990, 339655, 440932, 440960, 441001, 446974, 446983, 505197, 509472, and 559300) had significantly fewer eggs per gram of root than the susceptible check cv. Pickett 71, and fewer root galls (Fig. 1C–F). Among the accessions, all but PI 440960 had significantly fewer eggs per gram of root than the resistant check cv. Forrest. All the tested PIs except PIs 573045 and 559298 had lower gall indices than cv. Pickett 71. Only *G. tomentella* PIs 339655 and 446983 had a significantly lower gall index (Fig. 1C) than cv. Forrest.

**Table 2:** Eggs per gram of root and gall index of *Meloidogyne incognita* 8 weeks after inoculation.

***Glycine* species^a^**	**Accession**	**N^b^**	**Gall index^c,d^**	**Eggs per gram of root^c,e^**
*G. canescens*	PI 573045	9	6.7 a	34122 a
*G. latifolia*	PI 559298	7	6.1 a	13031 ab
*G. max* cv. Pickett 71 (S)	PI 548982	7	6.1 a	6896 ab
*G. microphylla*	PI 509487	9	4.1 b	40918 a
*G. tomentella*	PI 505238	10	3.0 bc	4285 ab
*G. clandestina*	PI 440960	6	3.0 b-d	285 c-f
*G. pescadrensis*	PI 505197	10	2.6 cd	52 ef
*G. tomentella*	PI 505214	10	2.4 cd	5058 ab
*G. cyrtoloba*	PI 509472	10	2.3 c-e	437 d-f
*G. latifolia*	PI 559300	8	2.3 c-f	210 d-f
*G. microphylla*	PI 505188	7	2.1 c-f	4597 ab
*G. canescens*	PI 440932	9	1.9 c-f	169 d-f
*G. tabacina*	PI 446974	10	1.8 c-f	430 de
*G. max* cv. Forrest (R)	PI 548655	10	1.8 c-f	1981 a-c
*G. argyrea*	PI 509451	5	1.7 c-g	579 b-d
*G. curvata*	PI 505167	6	1.2 d-h	4867 ab
*G. tabacina*	PI 373990	10	0.9 e-h	53 ef
*G. tomentella*	PI 441001	10	0.8 f-h	238 d-f
*G. tomentella*	PI 446983	9	0.3 gh	111 f
*G. tomentella*	PI 339655	10	0.2 h	3 f

Notes:

a S = susceptible check; R = resistant check.

b Sample number. The Bartlett tests for homogeneity of variances for eggs per gram of root and gall index were not significant (*P* > 0.05) between two trials so data were pooled before the analyses.

c Means with different letters are significantly different at *α* = 0.05 based on Tukey–Kramer HSD test.

d Bridge and Page, 1980.

e Eggs per gram of root were log (*x* + 1)-transformed before analysis and original data are presented here.

### Evaluation of perennial *Glycine* species for resistance to *R. reniformis*

The Bartlett test for homogeneity of variance was not significant (*P* > 0.05) between trials so data were pooled for analysis (Table 3). The perennial *Glycine* species and soybean checks differed in their response to *R. reniformis* based on the number of eggs and vermiform nematodes per gram of root. The mean number of *R. reniformis* per gram of root on susceptible check PI 88788 was significantly higher than that of resistant check cv. Forrest. In contrast to *M. incognita*, some perennial *Glycine* accessions (PIs 505188, 505214, 505238, 509487, 559298, and 573045) were significantly more susceptible to *R. reniformis* than the susceptible check PI 88788. Only *G. tomentella* PI 441001 had a significantly lower number of *R. reniformis* per gram of root than cv. Forrest.

**Table 3:** Number of eggs and vermiform *Rotylenchulus reniformis* per gram of root on perennial *Glycine* species and two soybean cultivars 10 weeks after inoculation.

***Glycine* species^a^**	**Accession**	**N^b^**	**Nematodes per gram of root^c^**
*G. canescens*	PI 573045	9	41245 a
*G. microphylla*	PI 509487	7	33424 a-c
*G. microphylla*	PI 505188	7	31744 ab
*G. tomentella*	PI 505238	10	31274 ab
*G. latifolia*	PI 559298	5	28578 a-c
*G. tomentella*	PI 505214	9	25039 a-c
*G. cyrtoloba*	PI 509472	8	10983 b-d
*G. tabacina*	PI 373990	6	10811 b-d
*G. pescadrensis*	PI 505197	10	8594 c-e
*G. canescens*	PI 440932	8	8265 c-e
*G. max* (S)	PI 88788	8	5910 d-f
*G. latifolia*	PI 559300	8	5897 d-f
*G. curvata*	PI 505167	8	5408 d-g
*G. tomentella*	PI 339655	7	2697 e-g
*G. tabacina*	PI 446974	9	1806 gh
*G. argyrea*	PI 509451	6	1783 f-h
*G. clandestina*	PI 440960	8	1511 i
*G. tomentella*	PI 446983	10	673 hi
*G. max* cv. Forrest (R)	PI 548655	10	306 i
*G. tomentella*	PI 441001	9	84 j
Fallow^d^		10	223^d^

Notes:

a S: susceptible check; R: resistant check.

b Sample number. The Bartlett test for homogeneity of variance was not significant (*P* > 0.05) between two trials so data were pooled before the analysis.

c Nematodes per gram of root was log (x) transformed before analysis and original data are presented. Means with different letters were significantly different at *α* = 0.05 based on Tukey-Kramer HSD test.

d Average of *R. reniformis* per pot from infested fallow soil control; fallow control was not included in analysis for final nematodes per gram of root.

### Evaluation of perennial *Glycine* species for resistance to *P. penetrans*

The Bartlett test for homogeneity of variance was significant (*P* < 0.05) between trials so data were analyzed separately (Table 4). For both trials, none of the perennial *Glycine* species showed reduced infection compared with the soybean varieties. For trial 1, *G. clandestina* PI 440960 and *G. tomentella* PI 339655 had significantly more nematodes per gram of root than cv. Lee 68, which had an average of 8 nematodes per gram of root. For trial 2, no accessions were significantly different from cv. Lee 68.

**Table 4:** Nematode per gram of root of *Pratylenchus penetrans* on perennial *Glycine* species and soybean cultivars six days after inoculation.

		**Trial 1^b^**		**Trial 2^b^**	
***Glycine* species^a^**	**Accession**	**N**	**Nematodes per gram of root^c^**	**N**	**Nematodes per gram of root^c^**
*G. clandestina*	PI 440960	5	259 a	4	349 a
*G. tomentella*	PI 339655	5	221 a	4	71 abc
*G. microphylla*	PI 505188	4	144 ab	2	124 abc
*G. tabacina*	PI 373990	4	96 ab	5	40 abc
*G. pescadrensis*	PI 505197	4	77 ab	4	111 abc
*G. tabacina*	PI 446974	4	70 ab	2	0 c
*G. tomentella*	PI 441001	4	65 ab	5	127 ab
*G. canescens*	PI 440932	5	34 ab	3	40 abc
*G. max* cv. Lee 68 (S)	PI 559369	5	8 b	2	64 abc
*G. max* cv. Williams 82	PI 518671	4	7 b	3	9 abc
*G. max*	PI 88788	4	7 b	5	2 c

Notes:

a Resistant soybean check is not available so not included in the test; S = susceptible check.

b N: sample number. The Bartlett test for homogeneity of variance was significant (*P* < 0.05) between two trials so analysis was done separately.

c Nematodes per gram of root was log transformed (x + 1) before analysis and original data are presented. Means with different letters are significantly different at *α*=0.05 based on Tukey-Kramer HSD test.

### Resistance rating

Of 18 PIs evaluated, 10 and 15 PIs were identified as resistant to *M. incognita* based on eggs per gram of root and gall index, respectively (Table 5) and three PIs were identified as resistant to *R. reniformis*, while PI 446974 was identified as moderately resistant. All of the eight perennial *Glycines* PIs evaluated for response to *P. penetrans* were identified as susceptible.

**Table 5:** Summary of the response of perennial *Glycine* species to *Meloidogyne incognita*, *Rotylenchulus reniformis*, *Pratylenchus penetrans*, *Heterodera glycines*, and *Phakopsora pachyrhizi*.

		***M. incognita^a^***		***R. reniformis^a^***	***P. penetrans^a^***	***H. glycines^b^***	***P. pachyrhizi^c^***
***Glycine* species**	**Accession**	**Eggs/g root^d^**	**Gall index^d^**	**Nematodes/g root^e^**	**Nematodes/g root^f^**		
*G. canescens*	PI 573045	S	S	S	-^g^	S	-
*G. latifolia*	PI 559298	S	S	S	-	-	-
*G. microphylla*	PI 509487	S	MR	S	-	S	-
*G. tomentella*	PI 505214	S	R	S	-	S	-
*G. microphylla*	PI 505188	S	R	S	S	R	-
*G. argyrea*	PI 509451	S	R	S	-	R	IM^h^
*G. curvata*	PI 505167	S	R	S	-	MS	-
*G. tomentella*	PI 505238	S	R	S	-	MR	-
*G. clandestina*	PI 440960	R	R	R	S	MR	S
*G. tabacina*	PI 446974	R	R	MR	S	S	R
*G. pescadrensis*	PI 505197	R	R	S	S	R	-
*G. latifolia*	PI 559300	R	R	S	-	-	-
*G. cyrtoloba*	PI 509472	R	R	S	-	R	-
*G. canescens*	PI 440932	R	R	S	S	R	-
*G. tabacina*	PI 373990	R	R	S	S	R	-
*G. tomentella*	PI 446983	R	R	R	-	S	-
*G. tomentella*	PI 441001	R	R	R	S	R	MR
*G. tomentella*	PI 339655	R	R	S	S	R	-

Notes:

a S (susceptible) ≥ susceptible check > MR (moderately resistant) > resistant check ≥ R (resistant). There was no MR for *M. incognita* eggs/g root since the susceptible check and resistant check were not significantly different. Rating was determined by the results from two tests for each of *M. incognita* and *R. reniformis*.

b MS = moderately susceptible to *H. glycines* HG 0 (Wen et al., 2017).

c Hartman et al., 1992; Herman et al., 2020.

d Eggs per gram root was determined as the total number of eggs extracted from roots per gram of fresh roots 8 weeks after inoculation. The gall index was determined by the extent of root galling comparing with the root-knot rating chart 8 weeks after inoculation (Bridge and Page, 1980).

e Nematodes per gram of root for *R. reniformis* was determined as the total number of eggs and vermiform nematodes from the roots and soil per gram of fresh roots 10 weeks after inoculation.

f Nematodes per gram of root for *P. penetrans* was determined as the total number of vermiform nematodes from the roots per gram of fresh roots 6 days after inoculation.

g Not tested or unknown.

h IM = Immune response to isolate MAL19 (Herman et al., 2020).

## Discussion

Soybean has narrow genetic diversity due to genetic bottlenecks (Hyten et al., 2006), while perennial *Glycine* species, wild relatives of soybean, have greater genetic diversity (Hwang et al., 2019). Transferring traits from perennial *Glycine* species to *G. max* by classical hybridization is challenging due to genetic barriers. Embryo rescue and colchicine treatment to produce amphidiploid plants (2*n* = 118) enabled hybridization between *G. ma*x cv. Dwight (2*n* = 40) and *G. tomentella* PI 441001 (2*n* = 78) (Akpertey et al., 2018; Singh, 2019). Hybrid lines with 2*n* = 40 and 41 chromosomes obtained by backcrossing with cv. Dwight showed resistance to soybean rust indicating successful genetic introgression of the disease resistance traits from PI 441001 to Dwight (Singh, 2019). Studies confirmed that perennial *Glycine* have novel sources of resistance to multiple SCN HG types (Wen et al., 2017; Herman et al., 2020). Our study shows that perennial *Glycine* species also have resistance to other soybean-parasitic nematodes including *M. incognita* and *R. reniformis* that infect and negatively affect yield in soybean. Finding novel resistance sources to additional nematode species in perennial *Glycine* species may lead to enhanced nematode resistance traits in soybean.

We evaluated 18 PIs from 10 perennial *Glycine* species for their response to *M. incognita* and *R. reniformis*, and eight PIs for response to *P. penetrans*. PIs were selected based on prior evaluation confirming a resistant or susceptible reaction to SCN (Wen et al., 2017), use in another genetic study (Chang et al., 2014), or due to availability of sequence information (Liu et al., 2018). Our results demonstrated that *M. incognita*, *R. reniformis*, and *P. penetrans* infected all PIs used in this study. *G. tomentella* PIs 441001 and 446983, and *G. clandestina* PI 446960 were classified as resistant to two nematode species, *M. incognita* and *R. reniformis*. Of these, PI 441001 was previously reported as resistant to SCN (Wen et al., 2017) and as moderately resistant to soybean rust (*Phakopsora pachyrhizi*) (Hartman et al., 1992). PI 440960 was reported as moderately resistant to SCN (Wen et al., 2017) and susceptible to *P. pachyrhizi* (Hartman et al., 1992). PI 446983 has not been identified as resistant to other pathogens. All PIs used in the *P. penetrans* tests were not significantly different from the susceptible check cv. Lee 68 and were thus classified as susceptible.

Several accessions were resistant or moderately resistant to *M. incognita* based on the gall index, but susceptible based on egg production (eggs per gram of root). This group included *G. argyrea* PI 509451, *G. curvata* PI 505167, *G. microphylla* PI 505188, PI 509487, *G. tomentella* PI 505214, and PI 505238. The contrast between reproduction and gall indices in these accessions to *M. incognita* was also previously seen in soybean (Harris et al., 2003); indeed, studies suggest that soybean QTL associated with *M. incognita* reproduction and root galling may be different (Tamulonis et al.,1997; Li et al., 2001; Ha et al., 2007; Fourie et al., 2008; Pham et al., 2013; Xu et al., 2013; Jiao et al., 2015; Passianotto et al., 2017; Li et al., 2018). Further investigation will be needed to understand the genetic basis for resistance in perennial *Glycine* species.

The observed range of responses in the perennial *Glycine* accessions to *M. incognita* or *R. reniformis* may be the result of the perennial *Glycine* accessions having independently developed resistance under selective pressure by these nematodes or may be due to other factors associated or genetically linked to resistance. Both *M. incognita* and *R. reniformis,* as well as *P. penetrans,* are found in Australia, Japan, Papua New Guinea and Taiwan where test accessions are native (Tu et al., 1972; Bridge and Page, 1984; Nakasono, 2004; Stirling, 2007; Hollaway et al., 2008; Min et al., 2011; Sherman-Broyles et al., 2014; Singh, 2019). A genomic study comparing a one million-base pair region in soybean with related legume species (including *G. tomentella*) found that, in contrast to conserved low-copy genes, gene families associated with disease resistance had undergone rapid diversification, such as genomic duplications and losses, and suggested that the rapid diversification of disease resistance genes might have been driven by pathogen-mediated pressure (Innes et al., 2008). Thus, even though the perennial *Glycine* species originate from the same geographical region, they may have undergone independent evolutionary events leading to variability in nematode resistance responses.

Our initial trials using pot-grown plants to infect the perennial *Glycine* species with *P. penetrans* were not successful in that we observed very low infection on plant roots 4 weeks after inoculation. Alternatively, using in vitro tests for *P. penetrans* and eight perennial *Glycine* species PIs that germinated and grew on MS medium supplemented with 2% sucrose, we observed either a similar or more susceptible response compared to cv. Lee 68 in all accessions, as well as in cv. Williams 82 and PI 88788, in both trials. One caveat to this in vitro test was that it only assessed infection and did not determine reproductive rates of *P. penetrans.* Though *P. penetrans* resistance has not yet been reported in soybean or perennial *Glycine* species, there have been previous reports of resistance or tolerance in soybean cultivars to other *Pratylenchus* species, for example, tolerance to *P. brachyurus* (Lindsey and Cairns, 1971), resistance to *P. scribneri* (Acosta and Malek, 1979), and moderate resistance to a new species of *Pratylenchus* spp. found in North Dakota (Chowdhury, 2020). Improvement of methods for *P. penetrans* infection and rating, as well as testing of additional PIs, is needed to determine if resistance exists in perennial *Glycine* species and soybean.

Perennial *Glycine* species identified in this study with resistance to *M. incognita* and *R. reniformis* may have novel nematode resistance genes not found in soybean. A genome-wide association study (GWAS) using wild soybean (*G. soja*) identified a novel SCN-resistance locus on chromosome 19 (Zhang et al., 2016). Beyond a recent success of hybridization between *G. max* cv. Dwight and *G. tomentella* PI 441001 (Singh, 2019), it may be possible to overcome the genetic barriers and transfer resistance genes from perennial *Glycine* to soybean using CRISPR-Cas9 gene-editing technologies (Sun et al., 2015). To increase the usefulness of genetic resistance found in perennial *Glycine* species and to discover and characterize additional resistance genes, molecular and genomic studies may provide the tools needed to further develop soybean resistance to *M. incognita*, *R. reniformis*, and *P. penetrans*. PIs identified in this study will serve as resources in ongoing efforts to identify novel nematode resistance genes for *M*. *incognita* and *R. reniformis*.
